# Heterologous COVID-19 Vaccination and Booster with mRNA Vaccine Provide Enhanced Immune Response in Patients with Cirrhosis: A Prospective Observational Study

**DOI:** 10.3390/vaccines11091455

**Published:** 2023-09-04

**Authors:** Pimsiri Sripongpun, Nawamin Pinpathomrat, Ratchanon Sophonmanee, Jomkwan Ongarj, Purilap Seepathomnarong, Bunya Seeyankem, Naichaya Chamroonkul, Teerha Piratvisuth, Apichat Kaewdech

**Affiliations:** 1 Gastroenterology and Hepatology Unit, Division of Internal Medicine, Faculty of Medicine, Prince of Songkla University, Songkhla 90110, Thailand; spimsiri@medicine.psu.ac.th (P.S.); naichaya.c@psu.ac.th (N.C.); teerha.p@psu.ac.th (T.P.); 2 Department of Biomedical Sciences and Biomedical Engineering, Faculty of Medicine, Prince of Songkla University, Songkhla 90110, Thailand; nawamin.p@psu.ac.th (N.P.); 6010210367@psu.ac.th (R.S.); 6310320007@psu.ac.th (P.S.); 6210320014@psu.ac.th (B.S.); 3 NKC Institute of Gastroenterology and Hepatology, Songklanagarind Hospital, Prince of Songkla University, Songkhla 90110, Thailand

**Keywords:** booster, cirrhosis, immune response, SARS-CoV-2, vaccination

## Abstract

This study aimed to evaluate the antibody and cellular responses to different coronavirus 2019 (COVID-19) vaccination regimens in patients with cirrhosis and to assess the antibody response after a vaccine booster. We conducted a prospective observational study of 89 patients with cirrhosis and 41 healthy volunteers who received two COVID-19 vaccine doses. Next, we prospectively evaluated 24 patients with cirrhosis who received a booster COVID-19 vaccine dose. In both studies, blood samples were collected before and 4 weeks after vaccination, and anti-spike receptor-binding domain protein IgG levels, T-cell phenotypes, and effector functions were assessed. The heterologous vaccine regimen (CoronaVac [SV]/AstraZeneca [AZ]) produced a better antibody response and CD4+IFNg+ T cell response compared to homogeneous vaccine regimens. The antibody response after the second dose of the vaccine was similar in patients with cirrhosis and healthy volunteers. Patients who received a booster dose of the mRNA vaccine had significantly increased antibody titers compared to those who received the AZ vaccine. In patients with cirrhosis, heterologous vaccination with SV/AZ resulted in a better immune response than the AZ/AZ and SV/SV regimens. Moreover, a booster dose of the mRNA vaccine led to a greater increase in antibody titers compared to the AZ vaccine.

## 1. Introduction

Severe acute respiratory syndrome coronavirus 2 (SARS-CoV-2), an emerging strain of coronavirus initially discovered in December 2019, is responsible for the occurrence of pneumonia outbreaks on a global scale [1]. The disease resulting from SARS-CoV-2 was designated the coronavirus disease of 2019 (COVID-19). As of August 2023, COVID-19 has affected at least 769 million people and has become a major public health problem worldwide [2]. In Thailand, more than 4.7 million cases have been confirmed, with a 0.7% mortality rate [2].

Patients with cirrhosis, particularly those with decompensated cirrhosis, have a greater risk of liver decompensation and an increased mortality rate associated with SARS-CoV-2 infection [3,4,5]. A multinational cohort study comprising 228 patients showed that among individuals with chronic liver disease but without cirrhosis, 43% experienced acute liver injury subsequent to SARS-CoV-2 infection. Furthermore, it was shown that one-fifth of individuals diagnosed with compensated cirrhosis experienced the development of either acute-on-chronic liver failure (ACLF) or acute decompensation. Liver-related complications were observed in almost half of the patients with decompensated cirrhosis, who tended to have more severe complications and a higher mortality rate [6]. Therefore, prevention of SARS-CoV-2 infection in patients with cirrhosis is imperative.

Many types of vaccines have been approved and are widely used, with efficacies ranging from 83.5% to 95% [7]. These include Moderna mRNA-1273, Pfizer/BioNTech BNT162b2 mRNA (PZ), the AstraZeneca/University of Oxford ChAdOx1-nCoV-19 chimpanzee adenovirus vector vaccines (AZ), and CoronaVac inactivated vaccine (SV) [8,9,10,11]. In general, the efficacy was high for mRNA and viral vector vaccines and moderate for inactivated vaccines. Nevertheless, there is a scarcity of data about the efficacy of various vaccination types in individuals diagnosed with cirrhosis.

A recent study of liver transplant recipients who received the PZ vaccine demonstrated only 47.5% positive immunogenicity after complete vaccination [12]. However, in general, in patients with cirrhosis who are not liver transplant recipients and are not on immunosuppressive agents, it remains controversial whether cirrhosis alone is associated with a lower immunogenicity after COVID-19 vaccination compared to that of the general population.

Moreover, Omicron is an emerging variant of SARS-CoV-2 that causes higher mortality rates than the wild type SARS-CoV-2. Two-dose vaccine regimens may not produce an adequate immune response against this novel strain; thus, a third booster dose may be required, particularly in immunocompromised patients.

Currently, no study has evaluated the humoral and cellular immune responses and neutralizing antibodies against Omicron after heterologous SARS-CoV-2 vaccination in patients with cirrhosis. Hence, the objective of our research was to assess the immunological response following the administration of two and three doses of COVID-19 immunization in individuals with cirrhosis who were given distinct types of booster vaccines, namely AZ and mRNA vaccines (specifically BNT162b2 Pfizer-BioNTech or mRNA-1273 Moderna vaccine).

## 2. Materials and Methods

### 2.1. Participants and Study Design

This prospective cohort study was conducted at Songklanagarind Hospital, a university tertiary hospital in southern Thailand. Patients with cirrhosis aged ≥18 years who planned to receive two doses of a standard COVID-19 vaccine regimen were recruited between June and October 2021. Liver cirrhosis was diagnosed on the basis of imaging or histological findings. The exclusion criteria were a prior history of symptomatic COVID-19, other immune deficiency syndromes (including congenital or acquired immune deficiency), pregnancy, a known vaccine allergy, and having not received two doses of vaccination.

The vaccination protocol given to the patients was according to the national policy of the Ministry of Health at that time. In general, patients would receive either SV/SV, AZ/AZ, or SV/AZ. During this study period for the first two doses, the mRNA vaccines were not widely available in Thailand and were not provided for the general population by the government. 

Baseline demographic data, including but not limited to age, sex, body mass index (BMI), cirrhosis etiology, Child-Turcotte-Pugh (CTP) score, comorbidities, presence of HCC within 6 months, liver chemistry test results, alpha-fetoprotein (AFP) levels, complete blood count, and international normalized ratio (INR), were collected.

The primary endpoints were antibody levels, cellular responses, and neutralizing antibodies after two COVID-19 vaccine doses in enrolled cirrhotic patients. In the primary analysis, we also compared the antibody levels of patients with cirrhosis to those of healthy volunteers. The age-matched (in the range of ±5 years) healthy volunteers (in a 1:2 ratio) were retrieved from the database of healthy volunteers who participated in another study conducted at our center at the same time. Healthy volunteers were defined as those aged ≥18 years without any underlying disease. The secondary endpoint was symptomatic SARS-CoV-2 infection after either the first or second dose until 31 July 2022.

Subsequently, the cirrhotic patients in the cohort who received a booster dose of vaccination between November 2021 and April 2022 and had no history of symptomatic COVID-19 between the vaccine doses were then invited to have the antibody level testing again after the 3rd dose of vaccine to evaluate the post-booster antibody levels, which were compared between the two types of booster vaccines—AZ and mRNA.

### 2.2. Laboratory Assays

#### 2.2.1. Blood Samples

Blood samples (15 mL) were collected before vaccination (on the same day as the first vaccination dose) and 4 ± 1 weeks after the second vaccination. Blood samples were separated into one clotted blood tube and two heparinized tubes. Heparinized blood was centrifuged according to the peripheral mononuclear cell (PBMC) separation protocol. The PBMCs were stored in liquid nitrogen for T-cell analysis, while the plasma samples as well as serum from clotted blood were kept at −80 °C. The serum was analyzed for antibody production. We also collected post-booster blood samples (4 ± 1 weeks after receiving a booster dose vaccination) to evaluate the antibody responses.

#### 2.2.2. SARS-CoV-2 Anti-Spike Receptor-Binding Domain Immunoglobulin G

The Abbott SARS-CoV-2 IgG II Quant assay was used to measure anti-receptor-binding domain protein (RBD) immunoglobulin G (IgG) responses. The method for the quantitative measurement is an indirect chemiluminescence immunoassay (CLIA). Results > 50 AU/mL were considered positive. Based on the WHO binding antibody unit (WHO BAU/mL), the conversion factor of 0.142 × AU/mL was equivalent to the WHO BAU/mL unit.

#### 2.2.3. SARS-CoV-2 Neutralizing Antibody

Thirty-six blood samples post complete two-dose vaccination were randomly selected for a 50% plaque reduction neutralization test (PRNT_50_) against Omicron BA.2. The PRNT was carried out at the Institute of Biological Products at the Department of Medical Sciences. The experiment was performed as described previously [13]. In short, Vero cells were seeded and incubated at 37 °C for 1 day. The neutralization was performed by mixing an equal volume of diluted serum and the diluted SARS CoV-2 virus in a water bath at 37 °C for 1 h. The virus-serum antibody mixture was inoculated into monolayer cells and incubated at 37 °C for 7 days. After the co-culture, the cells were fixed and stained with 0.5% crystal violet in PBS. The number of plaques was counted in triplicate wells and calculated as a percentage of plaque reduction at 50% (PRNT_50_).

#### 2.2.4. SARS-CoV-2 T-Cell Function

T-cell function was investigated using S1 peptide restimulation and flow cytometry. Flow cytometry staining was performed on cryopreserved PBMCs as described previously [14]. PBMCs were seeded and stimulated with the S1 peptide pool (ProImmune). Cells were incubated at 37 °C with 5% CO_2_. GolgiPlug (BD) was added after 2 h of incubation, and the culture was continued for another 16 h. After the ex-vivo restimulation, Live/Dead staining was performed for 10 min, followed by surface staining for 30 min of incubation. After the surface staining, the cells were fixed and permeabilized before staining intracellularly for 30 min at 4 °C. All the fluorochrome-conjugated antibodies were used as per Table S1. The stained cells were analyzed on a CytoflexS Beckman. The acquired data were analyzed using FlowJo Software (Version 10).

### 2.3. Statistical Analysis

To calculate the sample size, we used the preliminary results from the Center of Excellence in Clinical Virology at the Faculty of Medicine, Chulalongkorn University, Bangkok, which showed a 99% antibody response after administration of the complete inactivated vaccine (CoronaVac) in healthy volunteers [15]. We hypothesized that the antibody response rate following CoronaVac (55%) or AstraZeneca (75%) vaccine administration would be lower in patients with cirrhosis than in healthy volunteers. A sample size of at least 80 patients with cirrhosis was required at an alpha level of 0.05.

For statistical analyses, descriptive statistics are used for baseline characteristics data, in which continuous variables, e.g., age and antibody titers, are expressed as mean (±SD) or median (interquartile range [IQR]), according to the distribution of the data, and categorical variables are expressed as numbers (%). Comparisons between groups for the relevant outcomes were made using the *t*-test, Wilcoxon rank-sum test, and Fisher’s exact test. The statistical analyses were conducted using R software, version 4.1.0, developed by the R Foundation in Austria, as well as GraphPad Prism 9 software, version 9.4.0, developed by GraphPad Software Inc. A two-sided statistical significance level of *p* < 0.05 was deemed statistically significant.

### 2.4. Compliance with Ethical Standards

Prior to participating in the trial, all patients, including healthy volunteers, obtained informed consent. This study protocol received approval from the institutional review board of the Faculty of Medicine, Prince of Songkla University, Thailand (REC: 64-269-14-1). The research was carried out in compliance with the ethical principles outlined in the 1975 Declaration of Helsinki.

## 3. Results

### 3.1. Baseline Clinical Characteristics

During this study period, 116 cirrhotic patients were screened, of whom 95 were deemed eligible. Twenty-one patients were excluded because they eventually did not receive COVID-19 vaccination (*n* = 17), had concomitant corticosteroid use (*n* = 1), or had symptomatic COVID-19 (*n* = 3) before completing two doses of COVID-19 vaccines. Of the 95 eligible patients with cirrhosis, 42 age-matched healthy volunteers (the control group) were then identified. A total of 137 participants were eligible.

Of the 137 participants, the majority of vaccine regimens were AZ/AZ, followed by SV/AZ and SV/SV. Five patients in the cirrhosis group and one healthy volunteer received the vaccine regimen other than the three mentioned above (Cirrhosis group: 2 AZ/PZ, 2 Sinopharm/Sinopharm, and 1 PZ/PZ, Control group: 1 AZ/PZ), and therefore the data were too small to be representative and were then excluded from further analysis. Additionally, one patient in the cirrhosis group did not undergo the blood test after the second dose of vaccine; thus, the post-second dose vaccination titer cannot be determined. Finally, a total of 130 participants were included in the analysis.

The baseline clinical characteristics of the healthy volunteers (control group, *n* = 41) and the cirrhosis group (*n* = 89) are presented in Table S2 (Supplementary File). The median ages were 63 and 64 years, with 41.5% and 60.7% being male in the cirrhosis group and control group, respectively. The predominant vaccine regimen was AZ/AZ, followed by SV/AZ and SV/SV, with similar proportions between the two groups (*p* = 0.360).

Table 1 displays the baseline characteristics and antibody responses of patients in the cirrhosis group who received two doses of the COVID-19 vaccine. The data are stratified based on the vaccine regimens: AZ/AZ, SV/AZ, and SV/SV. Sex, cirrhosis etiologies, comorbid diseases, CTP scores, the proportion of patients with HCC within 6 months, and laboratory results were similar among the three vaccine regimens. Most patients were classified as CTP class A, followed by class B. There were only two CTP class C patients in the cohort: one in the AZ/AZ group, and the other one received the SV/AZ regimen. 

### 3.2. Comparison of Antibody Levels between Patients with Liver Cirrhosis and Healthy Volunteers

**Figure 1:**
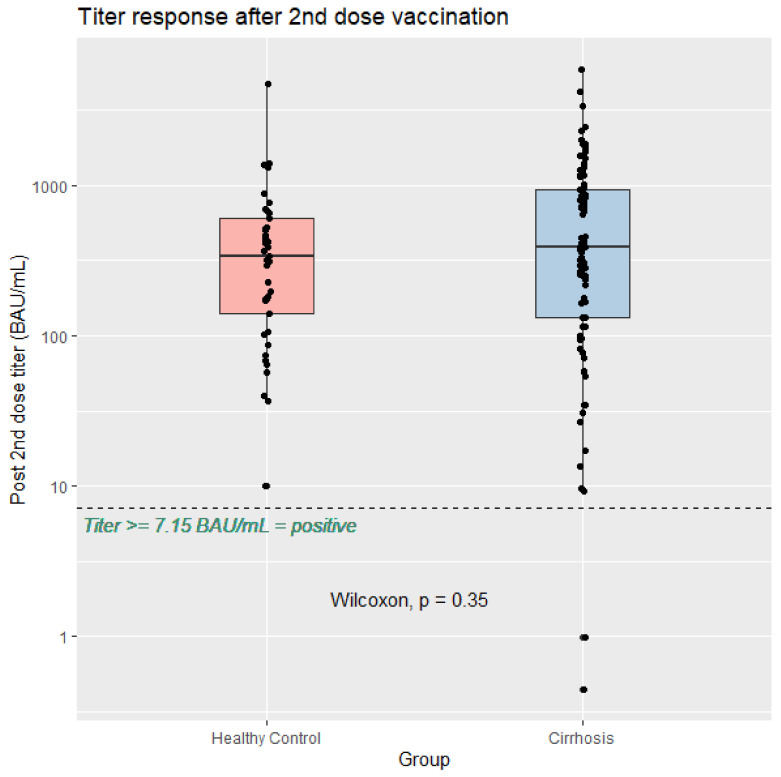
Serological response after the second dose of SARS-CoV-2 vaccination in patients with cirrhosis (*n* = 89) compared with healthy volunteers (*n* = 41).

When compared with healthy volunteers, similar antibody responses, referred to as anti-spike RBD IgG levels at week 4 after two vaccine doses, were observed between patients with cirrhosis and healthy volunteers (*p* = 0.35), as shown in Figure 1. Two patients (2.2%) in the cirrhosis group had anti-spike RBD IgG levels below 7.15 BAU/mL, which were considered to be vaccine non-responders, whereas none of the healthy volunteers was a non-responder; however, the proportion of non-responders between the two groups was not significantly different. 

### 3.3. Anti-Spike RBD Antibody in Liver Cirrhosis among the Three Regimens

**Figure 2:**
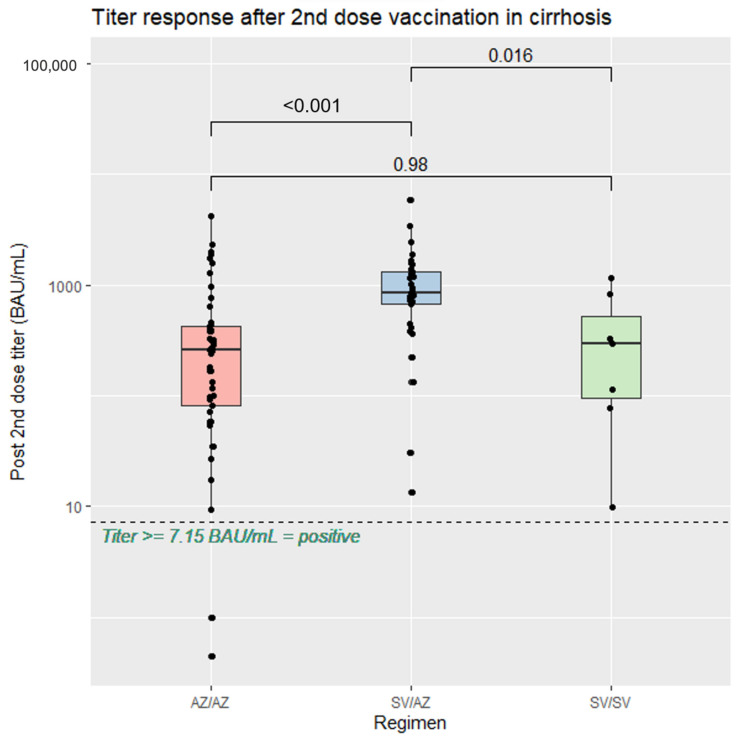
Serological response after the second dose of SARS-CoV-2 vaccination in patients stratified with cirrhosis according to type of vaccine.

**Figure 3:**
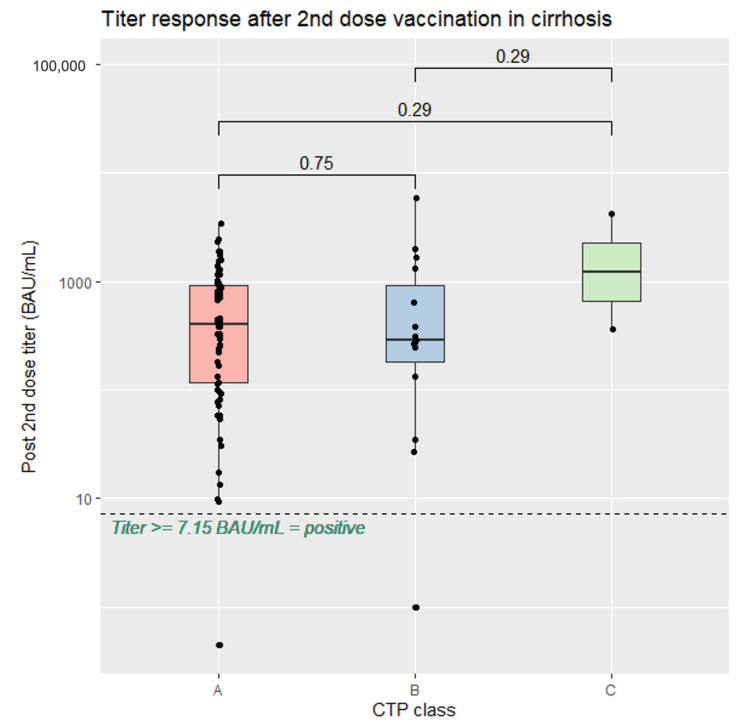
Serological response after the second dose of SARS-CoV-2 vaccination in patients with cirrhosis stratified according to CTP class.

In the comparisons between vaccine regimens, heterologous SV/AZ had the highest anti-spike RBD IgG level at a median level of 843.7 (IQR: 671.9, 1305.2) BAU/mL, significantly higher than AZ/AZ (median 258.2 [IQR: 81.2, 420.9] BAU/mL, *p* < 0.001) and SV/SV (median 295.7 [IQR: 95.8, 576] BAU/mL (*p* = 0.016), as shown in Figure 2. Among the patients with cirrhosis, similar antibody responses were observed across all CTP classes (Figure 3).

### 3.4. Assessment of Neutralizing Antibodies against the Omicron Variant following Different Vaccination Regimens

**Figure 4:**
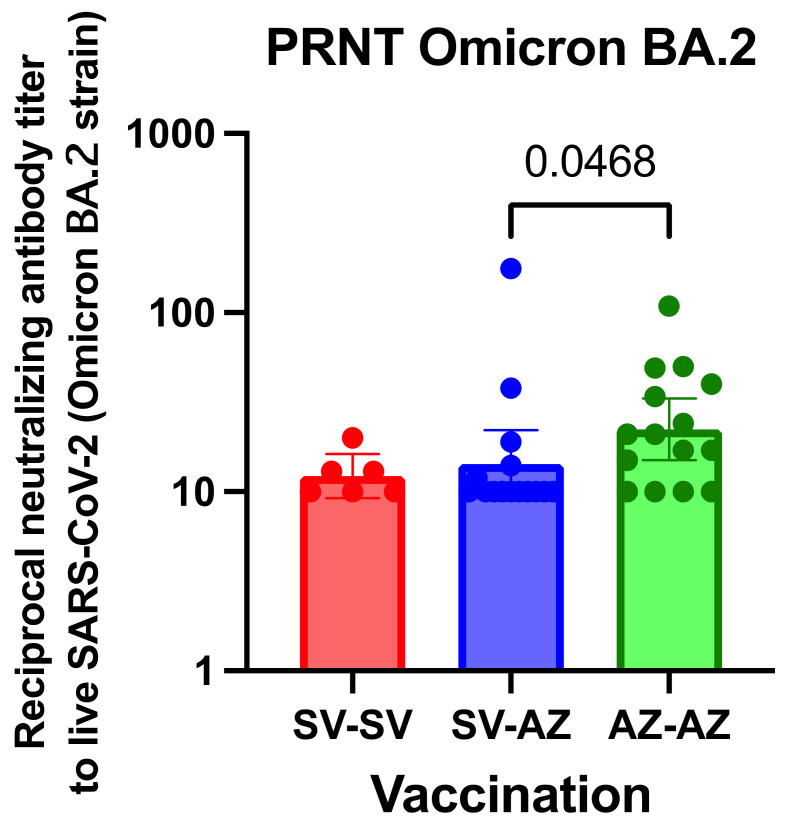
Neutralizing antibody against Omicron variant among the three regimens. Each symbol indicates the median value for an individual, with a 95% confidence interval (CI). Statistical evaluations were performed using the Kruskal-Wallis test, followed by post-hoc Dunn’s analysis for pairwise comparisons among the vaccine groups.

The 50% PRNT was employed to determine the inhibitory efficacy against the infectivity of the Omicron BA.2 variant. At least four weeks after receiving the second dose of the vaccine, there was a noticeable decline in the neutralizing potential of the antibodies produced (Figure 4). When comparing the neutralization efficacy against Omicron BA.2, those who received the AZ/AZ regimen exhibited a notably higher PRNT than those with the SV/AZ combination (*p* = 0.0468).

### 3.5. Cellular Immune Response in Liver Cirrhosis among the Three Regimens

**Figure 5:**
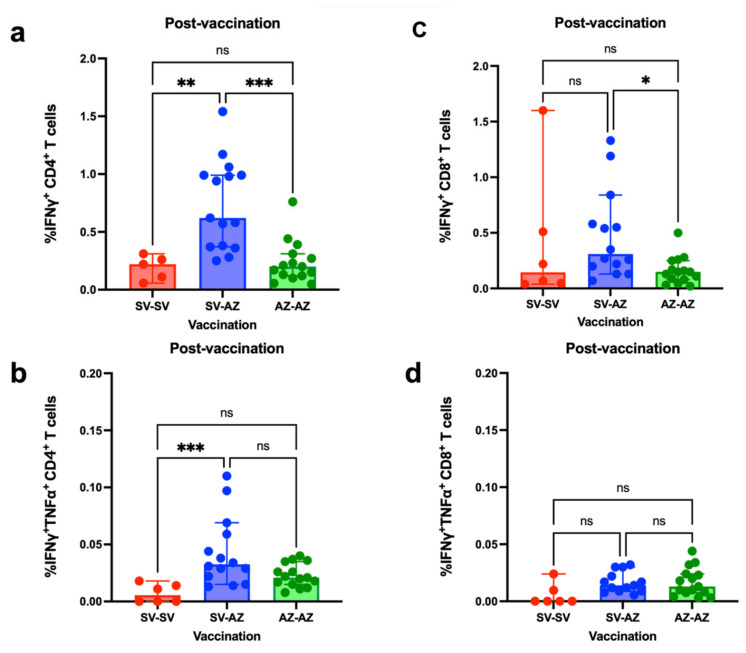
T-cell responses following the second dose of SARS-CoV-2 vaccination in patients with cirrhosis, categorized by vaccine type. (**a**) Proportion of CD4+ T cells eliciting IFN-γ production. (**b**) Proportions of CD4+ T cells releasing both IFN-γ and TNF-α. (**c**) Proportion of CD8+ T cells inducing IFN-γ secretion. (**d**) Proportions of CD8+ T cells generating both IFN-γ and TNF-α. Each symbol indicates the median value for an individual, with a 95% confidence interval (CI). Statistical evaluations were performed using the Kruskal-Wallis test, followed by post-hoc Dunn’s analysis for pairwise comparisons among the vaccine groups. * *p* ≤ 0.05; ** *p* ≤ 0.01; *** *p* ≤ 0.001.

T-cell responses revealed that the CD4+ IFN-γ + spike-specific T-cell response was highest in the SV/AZ group, and this was significantly greater than that in the SV/SV (*p =* 0.009) and AZ/AZ (*p* = 0.0004) groups (Figure 5a). Similarly, the SV/AZ group demonstrated a significantly higher presence of polyfunctional CD4+ T cells that secreted both IFN-γ and TNF-α compared to those in the SV/SV group (*p* < 0.0009), and this trended toward higher levels than those in the AZ/AZ group (Figure 5b). CD8+ T-cell responses exhibited a similar pattern, as significantly elevated IFN-γ producing CD8+ T-cell levels were observed in the SV/AZ group relative to those in the SV/SV group (*p* < 0.0264) (Figure 5c). Polyfunctional CD8+ T cells in the heterologous SV/AZ regimen were comparable to those in the AZ/AZ group but tended to be higher than those in the SV/SV group (Figure 5d). 

### 3.6. Post-Boost Anti-Spike Antibody Response

A total of 24 patients with cirrhosis who received their third dose of vaccination during the study period were enrolled. Among them, nine patients received the AZ vaccine, and 15 received the mRNA vaccine as a booster dose. Table 2 displays the baseline characteristics of those boosted with AZ and mRNA vaccines. No significant differences in baseline characteristics were observed between the two groups. The entire cohort had a mean age of 62.2 ± 13.5 years, with 58% being male, a median BMI of 25 kg/m^2^, hepatitis B as the most common etiology of cirrhosis, and 75% with CTP class A. 

**Figure 6:**
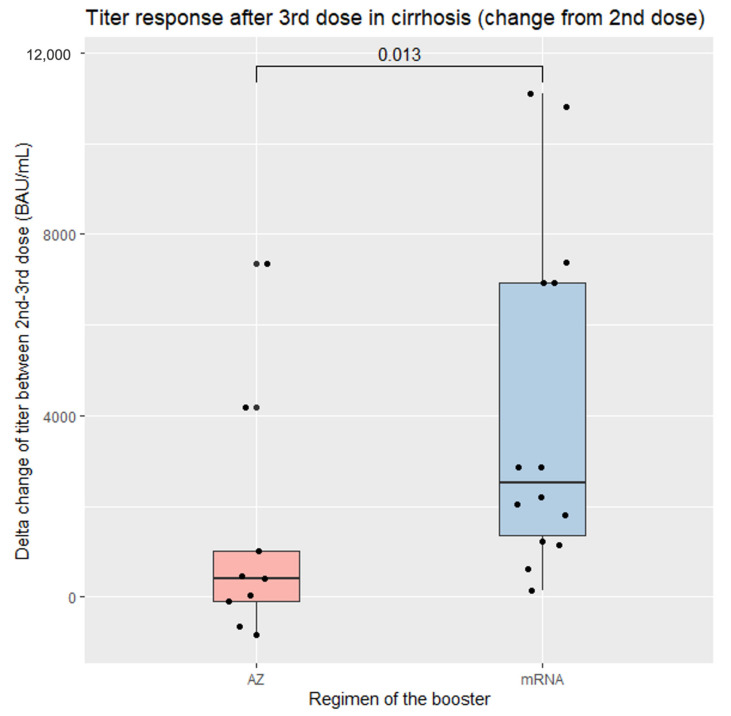
Serological responses after the booster dose of SARS-CoV-2 vaccination in patients with cirrhosis stratified according to booster vaccine.

Table 2 also presents the antibody responses of the two groups. After the booster dose, a trend towards higher mean antibody titers was observed in the mRNA group compared to the AZ group; however, statistical significance was not achieved. Changes in antibody titers (between pre-booster [after the second dose] and post-booster) were examined. Those who received the mRNA vaccine exhibited a significantly greater increase in antibody titers compared to those who received the AZ booster, with median levels of 2523.7 (IQR: 1365.6–6924.5) vs. 398 (IQR: −87.8–1015.6) BAU/mL (*p* = 0.015), respectively (Figure 6). No significant difference was observed in the primary vaccine regimen between the two groups.

### 3.7. Symptomatic SARS-CoV-2 Infection after Standard and Booster Vaccination

Three patients (SV/SV = 1, AZ/AZ = 1, and SV/AZ = 1) developed mildly symptomatic COVID-19 after the second dose. As of 30 June 2022, three patients had symptomatic COVID-19 following the booster dose (two patients in the AZ group and one in the mRNA group). All patients presented with mild symptoms without pneumonia, and none required hospitalization. No significant adverse events were reported in any patient after the booster dose.

## 4. Discussion

This prospective study demonstrates the similar antibody responses between patients with cirrhosis and healthy controls, we also highlighted the higher immunogenicity of both antibodies and cellular responses to the heterologous COVID-19 vaccine regimen in patients with cirrhosis. Additionally, a booster with an mRNA vaccine provided a better antibody response. This finding would be beneficial to encourage patients with cirrhosis, particularly those in Asian countries who have received a mixed vaccine platform, to obtain a booster vaccine with an mRNA vaccine.

The first finding was that antibody responses to the COVID-19 vaccination were comparable between patients with liver cirrhosis and healthy volunteers. This finding was similar to that of a meta-analysis showing that post-vaccination antibody responses did not differ between healthy controls and patients with chronic liver disease, including cirrhosis [16]. In contrast, a multi-center European study found that cirrhosis was a predictor of low immune response post COVID-19 vaccination, irrespective of etiology [17].

Cirrhosis-associated immune dysfunction (CAID) is a notable complication of cirrhosis that consists of two syndromic spectrums characterized by immune alterations: (i) a form of acquired immunodeficiency resulting from immunoparesis, and (ii) chronic systemic inflammation caused by immune system stimulation and disruption of homeostasis [7]. It has been hypothesized that CAID may contribute to poorer outcomes following SARS-CoV-2 infection in patients with decompensated cirrhosis and also a diminished immune response following vaccination in individuals with cirrhosis, especially in patients with decompensated cirrhosis, where CAID is more prevalent. Although an increased mortality in CTP class C cirrhosis patients with COVID-19 has been evidently reported [18], the data regarding a poor immune response following COVID-19 vaccination in patients with decompensated cirrhosis is still controversial. A study by Anand et al. demonstrated that antibody responses were clearly lower in people with decompensated cirrhosis than in healthy volunteers (non-responders: healthy controls 8% vs. decompensated cirrhosis 34%, *p* < 0.001) [19]. While in the current study, similar antibody titers were observed across the CTP classes, it is important to note that we included only a small number of patients with CTP class C (two patients; one received SV/SV and one received AZ/AZ). The results from our study are consistent with another study from India in which patients received two doses of Covishield (ChAdOx1nCoV-19) and similar antibody levels were found between patients with compensated and decompensated cirrhosis, with no correlation with CTP or Model for End-stage Liver Disease scores [20]. In a German study in which patients received mRNA vaccines, the median of SARS-CoV-2 IgG in patients with CTP class C cirrhosis was lower than that in CTP class A and B cirrhosis; however, without statistical significance (203 vs. 968 vs. 815 BAU/mL, in CTP classes C, A, and B, respectively) [21]. However, because a small number of CTP class C patients (*n* = 3) were included, the results should be interpreted with caution. 

Among the patients with cirrhosis, the heterologous SV/AZ vaccine regimen induced significantly higher anti-spike RBD IgG levels than the AZ/AZ or SV/SV regimens. Our study is the first study to demonstrate the efficacy of a mixed regimen of inactivated and adenovirus vector vaccines in patients with cirrhosis. This finding aligns with the results of a study showing that a heterologous inactivated prime vaccine followed by an adenovirus vector vaccine in healthy volunteers had a higher anti-RBD IgG in the SV/AZ group than in the SV/SV or AZ/AZ groups 4 weeks post vaccination (*p* < 0.001) [15]. In terms of cellular immune response, the SV/AZ group demonstrated the highest CD4+ IFN-γ + spike-specific T-cell response and polyfunctional CD4+ T cells, as well as elevated CD8+ T-cell responses. Enhanced immunity by the heterologous vaccine was supported by the finding from prime with the AZ vaccine, followed by the mRNA vaccine, with an increase in SARS-CoV-2 anti-spike IgG and cellular responses, more than that following the homologous vaccine (AZ/AZ) [22].

At the time of this study, Omicron BA.2 emerged, and we then explored the neutralized antibody after the 2-dose vaccine. This study represents the inaugural assessment of neutralizing titers against Omicron variants in individuals diagnosed with cirrhosis. Our study revealed that the neutralizing antibodies produced by heterologous SV/AZ vaccination regimens were found to be less effective compared to those generated by the AZ/AZ vaccination regimen. This finding was supported by the fact that a two-dose regimen with the inactivated vaccine did not produce sufficient immunity against the new Omicron variant [23]. Our results provide evidence to substantiate the necessity of administering a booster vaccine in response to the appearance of the Omicron variant.

We also explored the role of the booster vaccine (third dose) after receiving a 2-dose primary regimen. Our study highlights that a booster SARS-CoV-2 vaccine in patients with cirrhosis could enhance antibody responses, which were more pronounced when boosted with an mRNA than an AZ vaccine. According to the findings of Thuluvath et al., a proportion of 23% of individuals diagnosed with cirrhosis exhibited insufficient immune responses against SARS-CoV-2 subsequent to receiving either two doses of mRNA vaccines or a single dose of the Johnson & Johnson vaccine, which is an adenovirus vector-based vaccine typically provided as a single dose [24]. Similarly, the CHESS-NMCID 2101 study group revealed that 23.3% and 21.1% of compensated and decompensated patients with cirrhosis, respectively, had adequate neutralizing antibodies after two doses of inactivated vaccines [25]. Therefore, a booster vaccine may help enhance antibody responses in such patients.

Another finding of the present study was that the mRNA vaccine elicited better humoral immune responses than the AZ vaccine in patients with cirrhosis. This finding is similar to that of a study in healthy volunteers [26]. However, the healthy volunteer data came from a 2-dose vaccination, whereas our study used booster dose data. The same direction of antibody responses was found in a recent study from Brazil, in which the highest antibody levels were observed following the mRNA booster vaccine; however, that study had no data on patients with cirrhosis [27].

The strength of our study is that it is the first to demonstrate the immune effects of a heterologous primary regimen and booster dose of an AZ or mRNA vaccine in patients with cirrhosis. These findings have important implications for vaccination strategies in patients with liver cirrhosis, who are considered a vulnerable population. The use of a heterologous SV/AZ vaccine regimen could potentially provide better protection against COVID-19 for this group, and mRNA boosters may further enhance their immune response. Moreover, in scenarios where specific vaccine supplies are constrained, adopting a mix-and-match strategy can facilitate more adaptable vaccine distribution and administration. We also acknowledge the limited sample size of our study, as the booster dose vaccination was voluntary and it was inconvenient for some patients to commute to the hospital to receive blood tests during the pandemic. Further research is needed to corroborate these results and assess clinical outcomes such as the severity of SARS-CoV-2 infections, hospitalization rates, and mortality in patients with cirrhosis following vaccination. 

## 5. Conclusions

In conclusion, the antibody responses after two doses of COVID-19 vaccination were not different in patients with cirrhosis compared to those of healthy controls. The heterologous SV/AZ vaccination regimen seemed to induce a more robust T-cell response, both in terms of CD4+ and CD8+ T cells, compared to the homologous SV/SV regimen. The results also indicated that T-cell responses may be better following a heterologous SV/AZ regimen than a homologous AZ/AZ regimen, although the differences were less pronounced for some measures. These findings suggest that a heterologous vaccination strategy could be more effective in providing a better immune response against COVID-19, especially in settings where mRNA vaccines are not widely available. Additionally, patients with cirrhosis who received an mRNA booster vaccine showed a greater increase in antibody levels compared to those who received the AZ vaccine. Further research exploring which booster vaccines provide the best immune response against cirrhosis is required. 

## Figures and Tables

**Table 1:** Baseline characteristics and antibody responses after two doses of COVID-19 vaccination were stratified according to vaccine regimen among patients in the liver cirrhosis group (*n* = 89).

Baseline Characteristics	AZ/AZ (*n* = 49)	SV/AZ (*n* = 33)	SV/SV (*n* = 7)	*p*-Value
Age, years, mean (SD)	67 (63, 75)	61 (54, 68)	52 (45.5, 57.5)	<0.001
Male sex, no. (%)	32 (65.3)	18 (54.5)	4 (57.1)	0.634
BMI (kg/m^2^), median (IQR)	24.1 (22.2, 27.2)	24.2 (22.8, 26.2)	29 (26.4, 32.6)	0.025
Etiology, no. (%)				0.758
HBV	20 (40.8)	16 (48.5)	3 (42.9)	
Alcohol	10 (20.4)	5 (15.2)	0 (0)	
HCV	6 (12.2)	7 (21.2)	2 (28.6)	
NAFLD	5 (10.2)	4 (12.1)	1 (14.3)	
Cryptogenic	4 (8.2)	0 (0)	0 (0)	
AIH	3 (6.1)	0 (0)	0 (0)	
Other	1 (2)	1 (3)	1 (14.3)	
Comorbidity, no. (%)				
Diabetes	12 (24.5)	7 (21.2)	1 (14.3)	0.813
Hypertension	14 (28.6)	7 (21.2)	3 (42.9)	0.469
Renal failure	1 (14.3)	4 (12.1)	0 (0)	0.89
CTP class, no. (%)				0.527
A	37 (75.5)	28 (84.8)	7 (100)	
B	11 (22.4)	4 (12.1)	0 (0)	
C	1 (2)	1 (3)	0 (0)	
HCC within 6 mo, no. (%)	5 (10.2)	7 (21.2)	1 (14.3)	0.326
Laboratory				
TB, mg/dL, median (IQR)	0.8 (0.6, 1.2)	0.7 (0.5, 1)	0.8 (0.6, 0.8)	0.816
AST, U/L, median (IQR)	38 (29, 53)	39 (29, 49)	32.5 (24.5, 36.8)	0.268
ALT, U/L, median (IQR)	29 (24, 41)	27 (21, 41)	24.5 (22.5, 40)	0.768
ALP, U/L, median (IQR)	96 (76, 131)	88 (75, 135)	75 (54.5, 105.2)	0.292
Albumin, g/dL, median (IQR)	4.2 (3.5, 4.5)	4.2 (3.4, 4.5)	4.1 (4, 4.4)	0.732
WBC, cells/mm^3^, median (IQR)	5880 (3810, 6890)	5190 (4170, 6480)	7200 (6892.5, 7552.5)	0.098
Hb, g/dL, mean (SD)	12.7 (2.2)	12.7 (2)	14.3 (1.8)	0.208
Platelets, 10^9^/L, mean (SD)	140,020.4 (68,305.2)	142,030.3 (71,651.7)	172,000 (49,715.2)	0.559
INR, median (IQR)	1.1 (1.1, 1.3)	1.1 (1.1, 1.3)	1.1 (1.1, 1.2)	0.613
AFP, ng/mL, median (IQR)	3.7 (2.4, 5.3)	3.7 (2.3, 6.1)	2.4 (2.2, 6)	0.778
Antibody responses				
Pre-vaccination, BAU/mL, median (IQR)	0.3 (0.1, 0.6)	0.5 (0.1, 0.7)	0.2 (0, 0.3)	0.238
Post-vaccination 4 weeks, BAU/mL, median (IQR)	258.2 (81.2, 420.9)	843.7 (671.9, 1305.2)	295.7 (95.8, 576)	<0.001

AFP, alpha-fetoprotein; AIH, autoimmune hepatitis; ALP, Alkaline phosphatase; AST, aspartate aminotransferase; ALT, alanine aminotransferase; AZ, ChAdOx1-nCoV-19 vaccine (AstraZeneca and the University of Oxford); BAU, binding antibody units; BMI, body mass index; CTP, Child-Turcotte-Pugh; HBV, hepatitis B virus; HCC, hepatocellular carcinoma; HCV, hepatitis C virus; INR, International normalized ratio; NAFLD, nonalcoholic fatty liver disease; PBC, primary biliary cholangitis; SV, (CoronaVac or Sinovac); TB, total bilirubin.

**Table 2:** Baseline characteristics and antibody responses stratified according to the type of booster vaccine in patients with liver cirrhosis (*n* = 24).

Baseline Characteristics	AZ (*n* = 9)	mRNA (*n* = 15)	*p*-Value
Age, years, mean (SD)	65.6 (11.1)	60.1 (14.8)	0.353
Male sex, no. (%)	4 (44.4)	10 (66.7)	0.403
BMI (kg/m^2^), median (IQR)	23.7 (23.2, 26.7)	25 (24, 28.5)	0.456
Etiology, no. (%)			0.758
HBV	5 (55.6)	4 (26.7)	
Alcohol	1 (11.1)	3 (20)	
HCV	2 (22.2)	2 (13.3)	
NAFLD	5 (10.2)	4 (12.1)	
Cryptogenic	4 (8.2)	0 (0)	
AIH	3 (6.1)	0 (0)	
Other	1 (2)	1 (3)	
Comorbid disease, no. (%)			
Diabetes	1 (11.1)	1 (6.7)	1
Hypertension	4 (44.4)	2 (13.3)	0.15
Renal failure	2 (22.2)	1 (6.7)	0.533
CTP class, no. (%)			0.351
A/B/C	8 (88.9)/1 (11.1)/0	10 (66.7)/5 (33.3)/0	
HCC within 6 months, no. (%)	2 (22.2)	2 (13.3)	0.615
Laboratory			
TB, mg/dL, median (IQR)	0.6 (0.4, 0.7)	0.8 (0.7, 1.3)	0.816
AST, U/L, median (IQR)	32 (27, 36)	39 (27.5, 58)	0.282
ALT, U/L, median (IQR)	25 (24, 30)	29 (25.5, 47)	0.269
Albumin, g/dL, mean (SD)	4.2 (0.6)	3.8 (0.7)	0.232
WBC, cells/mm^3^, mean (SD)	6225.6 (2028.1)	5018 (1703.8)	0.132
Hb, g/dL, mean (SD)	13 (1.3)	12.8 (2.9)	0.812
Platelets, 10^9^/L, mean (SD)	181,111.1 (74,773.7)	125,200 (66,929.8)	0.071
INR, mean (SD)	1.1 (0.2)	1.3 (0.2)	0.119
AFP, ng/mL, median (IQR)	4.1 (2.3, 6)	3.3 (2.1, 4.9)	0.753
Primary regimen, no. (%)			0.217
AZ/AZ	2 (22.2)	9 (60)	
SV/AZ	5 (55.6)	4 (26.7)	
SV/SV	2 (22.2)	2 (13.3)	
Antibody responses			
Pre-booster, BAU/mL, median (IQR)	444.9 (238.3, 900.7)	324.8 (251.1, 728)	0.592
Post-booster 4 weeks, BAU/mL, mean (SD)	1960.5 (2434.1)	4566.8 (3706.7)	0.074
Delta titer, BAU/mL, median (IQR)	398 (−87.8, 1015.6)	2523.7 (1365.6, 6924.5)	0.013

AFP, alpha-fetoprotein; AIH, autoimmune hepatitis; ALP, Alkaline phosphatase; AST, aspartate aminotransferase; ALT, alanine aminotransferase; AZ, ChAdOx1-nCoV-19 vaccine (AstraZeneca and the University of Oxford); BAU, binding antibody units; BMI, body mass index; CTP, Child-Turcotte-Pugh; HBV, hepatitis B virus; HCC, hepatocellular carcinoma; HCV, hepatitis C virus; INR, International normalized ratio; NAFLD, nonalcoholic fatty liver disease; PBC, primary biliary cholangitis; SV, (CoronaVac or Sinovac); TB, total bilirubin.

## Data Availability

The data that support the findings of this study are available on request from the corresponding author.

## Supplementary Materials

The following supporting information can be downloaded at: https://www.mdpi.com/article/10.3390/vaccines11091455/s1, Table S1: Fluorochrome conjugated antibodies for flow cytometry analysis; and Table S2: Baseline characteristics and antibody responses after two doses of COVID-19 vaccine in the control group (*n* = 41) and liver cirrhosis group (*n* = 89).
