# Supplementary material for: Heterologous COVID-19 Vaccination and Booster with mRNA Vaccine Provide Enhanced Immune Response in Patients with Cirrhosis: A Prospective Observational Study
Source: Vaccines (Basel). 2023 Sep 4;11(9):1455. doi: 10.3390/vaccines11091455 (PMC10534824; doi:10.3390/vaccines11091455)
Supplement: Supplementary file 1 [file vaccines-11-01455-s001.zip › vaccines-2578771-supplementary.pdf]

Supplementary Table 1. Fluorochrome conjugated antibodies for flow cytometry analysis.

Antibody	Common Fluorochrome	Dilution	Clone	Source
**CD3**	PerCP	1:50	SP34-2	BD Horizon
**CD4**	APC-H7	1:200	SK3	BD
**CD8**	APC	1:200	SK1	BD
**L/D**	Aqua	1:1000	-	Invitrogen
**INF-Gamma**	PE-Cy7	1:100	B27	BD Pharmingen
**TNF-alpha**	PE-CF594	1:100	MAb11	BD Horizon

Supplementary Table 2. Baseline characteristics and antibody responses after two doses of COVID-19 vaccine in the control group (*N* = 41) and liver cirrhosis group (*N* = 89).

Baseline characteristics	Control group (*N* = 41)	Liver cirrhosis (*N* = 89)	*P* value
**Age, years, mean (SD)**	63 (53,66)	64 (58,72)	0.051
**Male sex, no. (%)**	24 (58.5)	35 (39.3)	0.064
**Vaccine regimen, no. (%)**			0.360
**AZ/AZ**	27 (65.9)	49 (55.1)	
**SV/AZ**	10 (24.4)	33 (37.1)	
**SV/SV**	4 (9.8)	7 (7.9)	

AZ, ChAdOx1-nCoV-19 vaccine (AstraZeneca and University of Oxford); SV, (CoronaVac or Sinovac)
